# Transmission of Engineered Plastids in Sugarcane, a C_4_ Monocotyledonous Plant, Reveals that Sorting of Preprogrammed Progenitor Cells Produce Heteroplasmy

**DOI:** 10.3390/plants10010026

**Published:** 2020-12-24

**Authors:** Ghulam Mustafa, Muhammad Sarwar Khan

**Affiliations:** Center of Agricultural Biochemistry and Biotechnology (CABB), University of Agriculture, University Road, Faisalabad, P.O. Box 38040, Pakistan; ghulam.mustafa@uaf.edu.pk

**Keywords:** plastid transformation, sugarcane, unfurled leaves, streptomycin, heteroplasmy, mesophyll and bundle sheath cells

## Abstract

We report here plastid transformation in sugarcane using biolistic transformation and embryogenesis-based regeneration approaches. Somatic embryos were developed from unfurled leaf sections, containing preprogrammed progenitor cells, to recover transformation events on antibiotic-containing regeneration medium. After developing a proficient regeneration system, the FLARE-S (fluorescent antibiotic resistance enzyme, spectinomycin and streptomycin) expression cassette that carries species-specific homologous sequence tails was used to transform plastids and track gene transmission and expression in sugarcane. Plants regenerated from streptomycin-resistant and genetically confirmed shoots were subjected to visual detection of the fluorescent enzyme using a fluorescent stereomicroscope, after genetic confirmation. The resultant heteroplasmic shoots remained to segregate on streptomycin-containing MS medium, referring to the unique pattern of division and sorting of cells in C_4_ monocotyledonous compared to C_3_ monocotyledonous and dicotyledonous plants since in sugarcane bundle sheath and mesophyll cells are distinct and sort independently after division. Hence, the transformation of either mesophyll or bundle sheath cells will develop heteroplasmic transgenic plants, suggesting the transformation of both types of cells. Whilst developed transgenic sugarcane plants are heteroplasmic, and selection-based regeneration protocol envisaging the role of division and sorting of cells in the purification of transplastomic demands further improvement, the study has established many parameters that may open up exciting possibilities to express genes of agricultural or pharmaceutical importance in sugarcane.

## 1. Introduction

Plastids have become attractive targets for genetic engineering since their genome offers several potential advantages, including accumulation of transporters to high levels with bona fide structures, biological containment of transgenes, gene stacking in operons, and absence of position effects [1,2]. Chloroplast genome has been engineered successfully to develop valuable traits, such as herbicide tolerance, insect and disease resistance, drought or salt tolerance, production of therapeutic proteins, antibodies, antibiotics, vaccine antigens, industrial enzymes, and other biosimilars in different plants [3,4,5]. Hence, more than 100 transgenes have been stably integrated and expressed in the chloroplast genome [6,7,8]. More recently, chloroplast genomes of major crops, including cotton, soybean, vegetables (carrot, lettuce, cabbage, eggplant, sugar beet), fruits (tomato), and trees (poplar and citrus), have been transformed [9,10].

Generally, monocots are recalcitrant to regeneration and there is no standard protocol available for efficient regeneration of plants from dedifferentiated cells, which could be used to transform plastids and to recover transgenic plastid-carrying cells. For each crop, different combinations of nutrients and auxins are used for callogenesis as well as organogenesis. To date, plastid transformation has been reported in rice, yet the regenerated shoots remained heteroplasmic at plastome, organelle, and cell levels [11,12]. One of the major bottlenecks in developing stable plastid transformation in monocots has been their regeneration from non-green embryonic cells, containing undifferentiated plastids. Other barriers in developing homoplasmic transplastomic plants, particularly of rice, might be the low level of marker gene expression in non-green plastids in embryogenic cells because of a low genome copy number and low rates of protein synthesis [13].

While addressing bottlenecks, we attempted to transform the sugarcane plastid genome where we developed an efficient regeneration protocol and species-specific chloroplast transformation vector carrying the dominant visual selectable marker. The development of transplastomic technology in sugarcane will open up exciting possibilities for novel gene introduction and expression for agricultural or pharmaceutical traits.

## 2. Results

### 2.1. Development of a Proficient Regeneration Protocol

**Figure 1:**
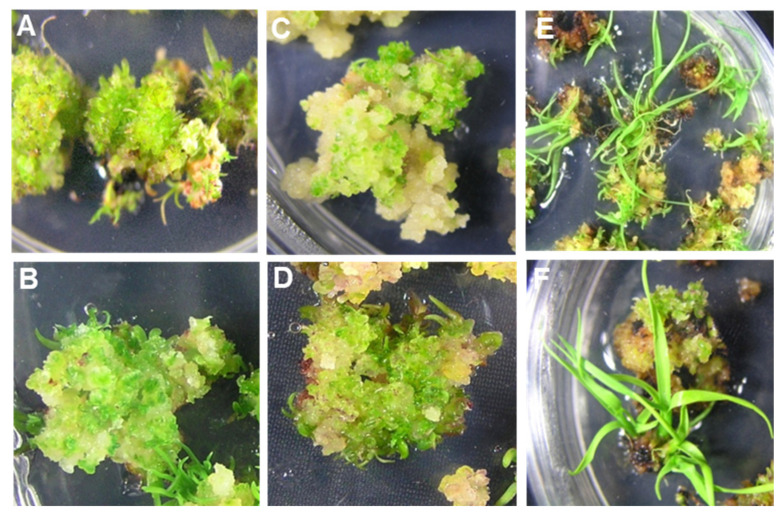
Efficient multiple shoot regeneration from embryogenic calli developed from undifferentiated young leaf whorls of different cultivars of sugarcane on the RMSDBK medium. (**A**) US-778 (**B**) HSF-240 (**C**) US-64 (**D**) CPF-246 (**E**) US-127 (**F**) HSF-242.

**Figure 2:**
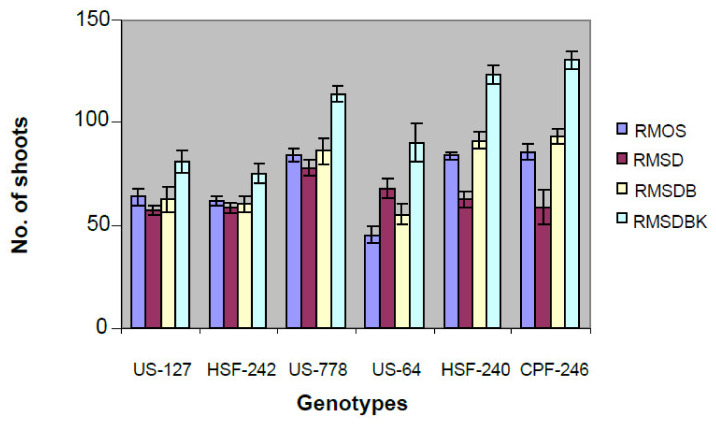
Effect of different types of media on shoot induction from calli derived from young leaf whorls of different genotypes. Most responsive genotype to regeneration was CPF-246 on RMSDBK medium.

Unfurled leaves from six field-grown elite sugarcane genotypes (CPF-246, US-127, HSF-242, US-778, HSF-240, and US-64) were sacrificed to develop leaf roll discs, and subsequently placed on callus induction medium. Of six genotypes, maximum embryogenic cells were recovered from genotypes US-127, US-778 on MS medium supplemented with 2,4-D (2,4-Dichlorophenoxyacetic acid) ranging from 3 to 5 mg/L. Five-week-old dark proliferated calli were subjected to many combinations of hormones (RMOS, RMSD, RMSDB, and RMSDBK) (Section 4.2) and the maximum number of shoots was observed on RMSDBK medium (Figure 1). However, it was observed that maintenance of 2,4-D in combination with kinetin and BAP (6-Benzylaminopurine) results in healthy and separable mature plants, contrary to earlier studies (Figure 2) [14,15]. BAP, kinetin, and 2,4-D had synergistic effects on shoot regeneration [16,17].

### 2.2. Development of Species-Specific Chloroplast Transformation Vectors

**Figure 3:**
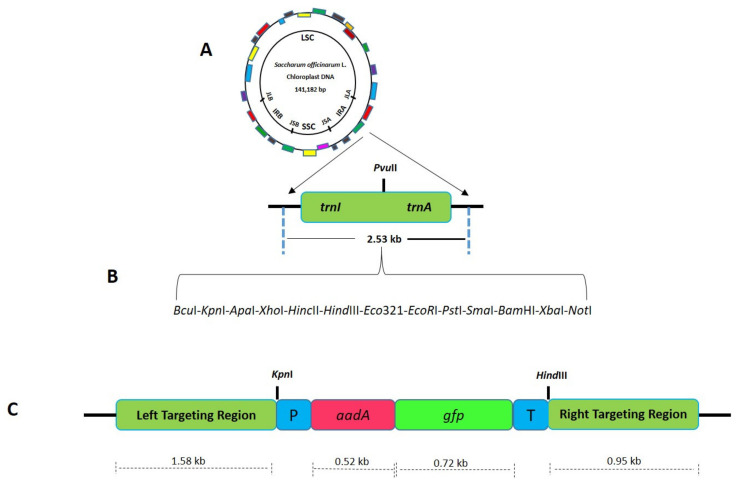
Schematic diagram showing the cloning strategy for the development of sugarcane plastid transformation vector SOFM2. (**A**) Sugarcane flanking sequences used for site-specific integration of transgene in the plastome inverted repeats. (**B**) Adapter sequence was cloned in between the flanking sequences to engineer multiple cloning sites. (**C**) Final sugarcane plastid transformation vector (SOFM2) with *aadA* and *gfp* genes.

After the establishment of a proficient regeneration system, a species-specific sugarcane plastid transformation vector was developed. Species-specific flanking sequences play a pivotal role in plastome engineering as a drastic decrease in transformation efficiency was observed when flanking sequences of petunia were used instead of tobacco for *Nicotiana* plastome transformation [18], suggesting that a lack of complete homology of the targeting sequences results in low transformation efficiency [19]. The stable plastid transformation system depends upon the integration of foreign DNA into the plastid genome through homologous recombination [20]. To facilitate homologous recombination, the inverted repeat region of sugarcane was amplified using primers P1 5′-GAT ATC AAA ACC CGT CCT CAG TTC GGA TTG C-3′ and P2 5′-GAT ATC CAC GAG TTG GAG ATA AGC GGA-3′. The *trn*I-*trn*A intergenic regions were selected to integrate transgene into the plastome since these regions have successfully been used to develop transgenic chloroplasts [21]. To engineer suitable restriction sites, for further cloning, an adapter sequence carrying restriction sites for *Bcu*I, *Kpn*I, *Apa*I, *Xho*I, *Hinc*II, *Bsu*15I, *Hind*III, *Eco*321, *Eco*RI, *Pst*I, *Sma*I, *Bam*HI, *Bcu*I, *Xba*I, *Not*I, *Bstx*I, and *Sac*I was cloned (Figure 3). The fluorescent selectable marker *FLARE-S* having *aadA* (aminoglycoside-3’-adenyltransferase) gene translationally fused with *gfp* (green fluorescent protein), was used to visually detect transformed cells since this is the most commonly used selection system giving out high transformation efficiency, developed by Khan and Maliga [11].

### 2.3. Plastid Transformation and Recovery of Transplastomic Plants

**Figure 4:**
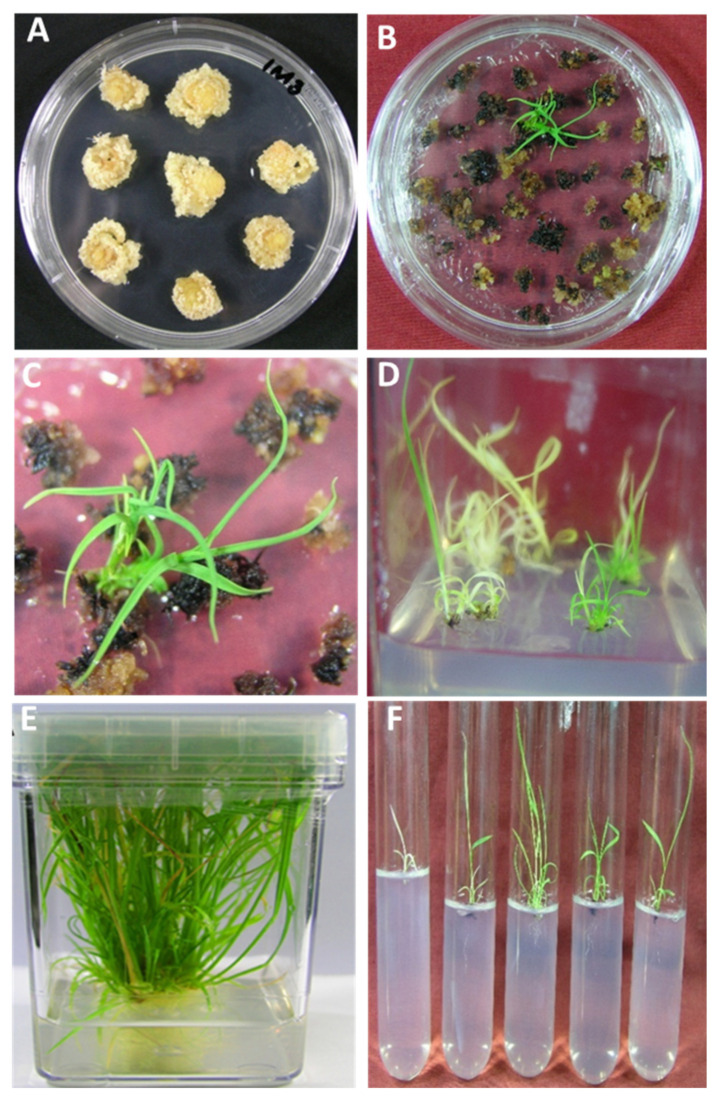
Purification of shoots carrying transformed plastomes on streptomycin-containing regeneration medium. (**A**) Calli proliferated under dark growth conditions were used to transform plastomes (**B**) Selection of cells, after the bombardment, on streptomycin-containing regeneration medium where resistant cells are regenerated into shoots while sensitive are bleached. (**C**) Close up of the resistant shoots as shown at B. (**D**) Root initiation on streptomycin-containing rooting medium. (**E**,**F**) Multiplication of regenerated shoots on MS medium in jars and glass tubes with and without IBA, respectively.

**Figure 5:**
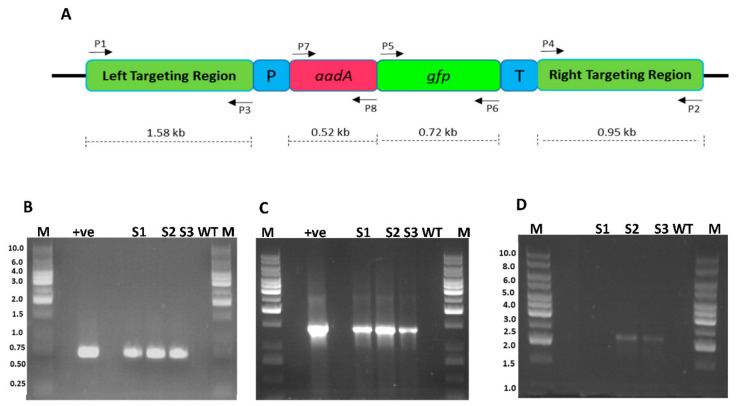
Confirmation of integration of FLARE-S cassette into the plastid genome of sugarcane. (**A**) Physical map of sugarcane plastid transformation vector showing the position of primers flanking various genes (**B**) PCR amplification of marker gene (*gfp*) with primer sets P5/P6: lanes M 1 kb DNA ladder, lane +ve shows amplification from plasmid DNA, lanes S1, S2, and S3 are transformed sugarcane plant DNA, lane WT untransformed sugarcane plant DNA (**C**) PCR amplification of FLARE-S with primer sets P6/P7: lanes M represent 1 kb DNA ladder, lane +ve represents plasmid DNA as template, lanes S1, S2, and S3 are transformed sugarcane plant DNA, lane WT untransformed sugarcane plant DNA (**D**) PCR amplification of left border sequence along with marker gene (*aadA*) with primer sets P1/P8: lanes S1, S2, and S3 are transformed sugarcane plant DNA, lane WT untransformed sugarcane plant DNA, lanes M 1 kb DNA ladder.

Selectable markers are essential tools for chloroplast transformation [11,22] and the only available dominant marker is *aadA*, conferring resistance to streptomycin and spectinomycin. Similar to other monocotyledonous cereals, sugarcane is naturally resistant to spectinomycin. Hence, streptomycin was used to select transformation events. After developing a kill curve, 300–350 mg/L streptomycin was determined as optimal for selection [23]. Three-week-old calli were bombarded using the PDS-1000/He biolistic gun (Bio-Rad, Hercules, CA, USA) following the established protocol [24]. The bombarded calli maintained on MS1.5 medium for 2–3 weeks were transferred onto streptomycin (350 mg/L)-containing RMSDBK medium where transformed cells proliferated and regenerated into shoots. However, wild-type calli turned brown and ultimately became dead (Figure 4). GFP facilitated the selection and screening of the transformants. Calli chunks fluorescing green under UV light were separated from non-fluorescent cells and were regenerated into shoots. The regenerated shoots were shifted to the rooting medium. The antibiotic-resistant primary regenerants showed a great degree of phenotypic segregation during selection. Variegation was also observed in proliferating leaves along with green shoots. The green shoots appeared to be positive for marker gene presence and integration into plastome (Figure 5), inferring that the variegated shoots were highly heteroplasmic and transgenic plastids were segregating at the cell as well as tissue levels.

### 2.4. Tracking Transgenic Plastids Using Green Fluorescence Protein (GFP)

**Figure 6:**
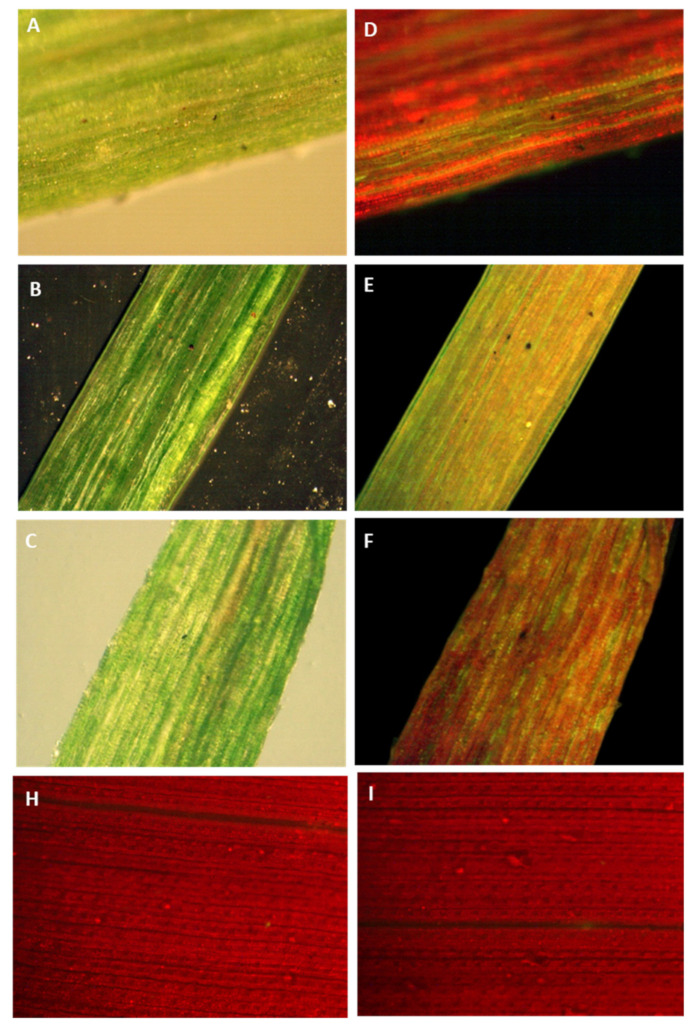
Tracking green fluorescent protein in sugarcane leaves by a stereomicroscope equipped with a GFP detection system. GFP fluoresces green whereas chlorophyll fluoresces red when exposed to fluorescent illumination. (**A**–**C**) Images of transformed sugarcane leaves in bright or dark field illumination. (**D**–**F**) Images of transformed sugarcane leaves in fluorescent illumination. (**H**–**I**) Images of untransformed sugarcane leaves in fluorescent illumination.

**Figure 7:**
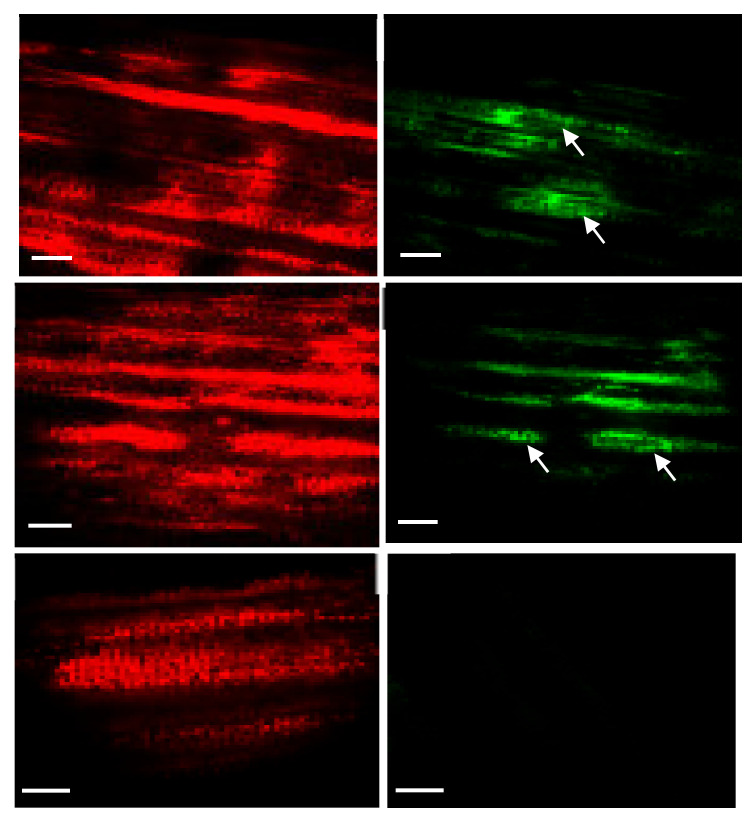
Visualizing fluorescent sectors in transformed sugarcane leaves under a laser scanning confocal microscope. The red channel and the green channel were imaged separately. Images were processed to detect chlorophyll (**A**–**C**) and Green Fluorescent Protein (**D**–**F**). Transplastomic plant leaf sectors fluoresced green (**D**,**E**) whereas no fluorescence was detected in untransformed sugarcane leaves (**F**) under the green channel.

**Figure 8:**
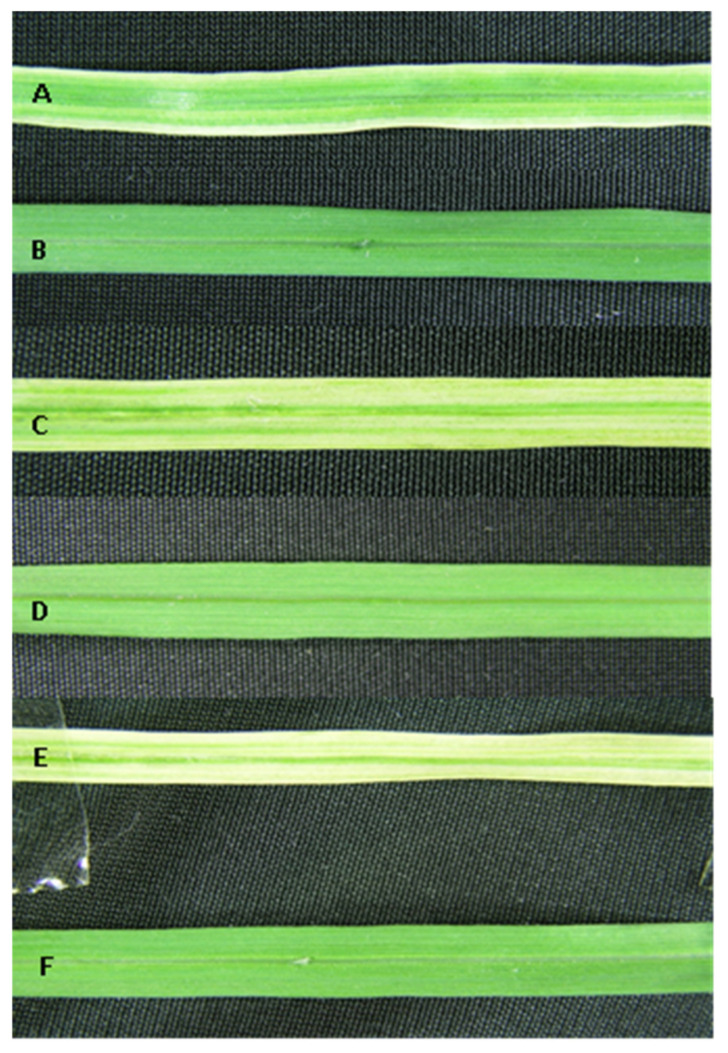
The phenotypic segregation of the antibiotic-resistant sugarcane transformants. Variegation was observed in leaves of plants growing on streptomycin-containing MS medium. (**A**,**C**,**E**) Variegated leaf segments of putative sugarcane transformants. (**B**,**D**,**F**) Lush green leaf segments of untransformed sugarcane plants growing on streptomycin-free medium in the same conditions.

The antibiotic-resistant calli and regenerated shoots were initially inspected for fluorescence using a hand-held long-wave UV lamp. The proliferating calli and leaves of the putative transplastomic sugarcane plants were subjected to a stereomicroscope, SZX (Olympus America, Melville, NY, USA) equipped with a GFP detection system. The green-fluorescing calli were selected and proliferated on streptomycin-containing MS medium supplemented with MS vitamins, 3 mg/L 2,4-D and were regenerated into shoots upon transfer to RMSDBK medium. Green-fluorescing sectors were observed in the leaves of antibiotic-resistant transgenic plants, confirming that the *gfp* gene was integrated into sugarcane plastome (Figure 6). These fluorescing sectors varied in size in leaves and even in different plants, depending upon the expression of the transgene as well as segregation of transplastomic and wild-type plastids (Figure 7). Such chimerism necessitates another cycle of regeneration on the selective medium. Accumulation of green fluorescent protein (GFP) was also assessed in the leaves of PCR-positive plants by confocal laser scanning microscopy. The FLARE-S expression confirmed the segregation of transplastomic and wild-type plastids in streptomycin-resistant sugarcane (Figure 8). It was observed that cells surrounding the veins are fluorescent, indicating chloroplasts in bundle sheath cells are only transformed, and regular files of these cell clones are extended in an aligned fashion.

### 2.5. Tracking Transgene Integration through PCR Approach

The putative transgenic plants were analyzed for marker gene(s) integration into the plastid genome using the PCR approach. Two gene-specific primers for *gfp* were used. Clones 3, 5, and 6 were found positive for *gfp* gene integration since a fragment of 721 bp was amplified (Figure 5B). Further, to reconfirm the presence of translationally fused genes (*gfp* and *aadA*), another set of primers where forward primer landed on the *aadA* gene and reverse primer on the *gfp* gene were used. Amplification of a fragment of 1452 bp from clones 3, 5, and 6 confirmed the integration of the transgene into the plant genome. These experiments only verified the presence of transgenes in the plant genome but did not authenticate whether transgene was integrated into the plastome. To verify whether the transgenes are integrated into the chloroplast genome, another pair of primers was designed where one primer lands on native plastome and other on transgene (*aadA*). The amplification of a fragment of 2259 bp confirmed their transplasmicity, thus eliminating the possibility of spontaneous mutants, escapees, or even nuclear transformants.

However, plastid transformation was achieved in sugarcane, but low transformation efficiency and heteroplasmy remained to be resolved. The transformation efficiency resulting in one transformation event per 27 bombarded plates was found to be lower than reported in *Arabidopsis* [25,26], potato [27,28,29], and tomato [30,31]. However, transformation efficiency was comparable with rice [11] since target tissues in both cases were embryogenic calli. Plastids in the dark-grown calli are normally undeveloped, having a size of ~1 μm (approximately 5 to 10-fold smaller than well-developed chloroplasts in the green leaf tissue, turning out to be tough targets for transformation with 0.6 μm gold/tungsten particles owing to increased physical damage. The use of smaller-sized metal particles (0.4 μm) is reported to improve the transformation efficiency of proplastids by three- to four-fold [32]. Another reason could be the levels of transcription and translation that is lower in proplastids than mature chloroplasts [33]. The strong constitutive promoter (*Prrn*) was used to drive transgene expression but its activity has been reported to be low in proplastids. Thus, promoters encompassing both the PEP (plastid-encoded RNA polymerase) and NEP (nucleus-encoded RNA polymerase) would be the promoters of choice to increase transgene accumulation in sugarcane proplastids [34]. Another major impediment in achieving homoplasmic clones is the anatomy of the sugarcane plant where mesophyll and bundle sheath cells originate from preprogrammed dividing cells, and these cells are aligned in a regular fashion in the leaves.

## 3. Discussion

Plastid genome engineering is extended to many plant species, including tobacco, potato, cotton, tomato, carrot, oilseed rape, petunia, sugar beet, lettuce, cabbage, eggplant, and soybean, owing to the unique advantages of the expression of transgenes in polycistronic units, accumulation of transporters to high levels with bona fide structures, biological containment of transgenes because of maternal inheritance, and elimination of position effects that are frequently observed in nuclear transformation because of random insertion of transgenes into the genome. Plastid genome engineering is still very incipient in C_3_ monocotyledonous crops, and it has not so far been reported for the C_4_ sugarcane crop. Hence, the plastid genome of sugarcane is engineered. While designing experiments, three major bottlenecks to achieve homoplasmic transgenic plants were considered: one, referring to regeneration, the recalcitrance was thought to be the major bottleneck in plastome engineering of monocotyledonous crops as no mature leaf-based regeneration system is available. Therefore, many genotypes were used to assess the regeneration of cells/shoots on different combinations of hormones [35,36]. Consequently, a proficient regeneration system with higher regeneration efficiency than earlier reports [24,37] was developed.

The only dominant available marker for selection of plastid transformation events is the *aadA* gene that encodes aminoglycoside 3′-adenyltransferase and confers resistance to spectinomycin and streptomycin. Spectinomycin has been used extensively for selecting the transformation events in different plants like tobacco, potato, lettuce, tomato, cabbage, sugar beet, and eggplant, where green heteroplasmic shoots were recovered due to the phenotypic masking effect of transformed cells, subsequently purified through additional rounds of selection and sorting out of cells. Unlike these plants, rice [11] and sugarcane [23] are naturally resistant to spectinomycin. So, the second bottleneck was the choice of an antibiotic for selection and screening of transformation events on regeneration medium; and the only choice left was streptomycin. Hence, streptomycin was used in subsequent experiments after developing a kill curve [23]. Using streptomycin, transplastomic plants were recovered on the regeneration medium.

Third, the major impediment to plastid genome engineering in sugarcane observed was the presence of two preprogrammed distinct types of cells, bundle sheath and mesophyll, developed upon differentiation of meristematic cells. In C_4_ plants, including maize, the bundle sheath (BS) lineage is distinct from that which produces mesophyll (M) cells [38]; this differentiation into the various cell types is driven by a complex interaction of transcription factors, small interfering RNAs, metabolites, and phytohormones [39]. Hence, the transformation of a proplastid in a non-differentiated or preprogrammed cell in the meristem tissue or calli may inherit transformed proplastid into a typical recipient cell type (either BS or M) and the transformed proplastids differentiate into chloroplasts leading towards heteroplasmy. This is because regular files of cell clones extend in an aligned fashion from the base to the tip of the leaf [40,41]. Emission of green fluorescence from aligned cells in leaves of regenerated streptomycin-resistant shoots (Figure 6) supports our hypothesis that a recipient cell of either BS or M upon transformation will divide and regular files of these cells will extend continuously in an aligned fashion in the leaves of a transgenic shoot. However, non-recipient cells will also grow similarly. Further, it is highly unlikely that transformed proplastids are inherited into both types of cells (mesophyll or bundle sheath), generating a homoplasmic plant. This, then, is one aspect that calls for further investigation.

## 4. Materials and Methods

### 4.1. Choice of Explant Material and Callus Induction

Various plant parts, including nodal tissues, apical meristem, the sub-apical region of the shoot apex, young leaf whorls, and root meristematic tissues, were used to develop calli and for proliferation. Release of phenolic compounds from all explants except young leaf whorls were a major problem in developing the calli and regeneration protocol, resulting in browning of tissues. Hence, the protocol for young leaf whorls was developed. Sugarcane tops of 6–8-month-old plants were sterilized using 70% ethanol and then sliced into transverse sections. Young leaf roll discs of 1.5–2.0 mm thickness and 60 to 95 mm^2^ area cross-section were used to develop on callus induction medium, having different levels of 2,4-D (1, 2, 3, 4, and 5 mg/L). A suitable level of 2,4-D was selected that was tested in combination with different levels of kinetin (0.5, 1.0, 1.5, 2.0, 2.5, 3.0 mg/L).

### 4.2. Shoot Induction, Multiplication, and Rooting

Embryogenic calli were transferred to the RMOS medium. RMOS consists of Murashige and Skoog salts supplemented with thiamine HCl 1.0 mg/L, nicotinic acid 0.5 mg/L, pyridoxin HCl 0.5 mg/L, glycine 2 mg/L, myoinositol 100 mg/L, casein hydrolysate 500 mg/L, and sucrose 30 g/L. Afterward, calli were transferred to RMOS supplemented with 2,4-D (RMSD), 2,4-D and BAP (RMSDB) and 2,4-D, BAP, and kinetin (RMSDBK). The plates were incubated in low light (1400 lux day intensity) following 16:8 h light: dark regime for one week, and then in bright light (2000–2500 lux day intensity) at 26 ± 1 °C. The media were solidified using 2.6 g/L phytagel with a pH of 5.7. For multiplication and robust rooting, regenerated plants were transferred to the MSV medium (Murashige and Skoog’s basal medium supplemented with IBA).

### 4.3. Construction of Species-Specific Chloroplast Transformation Vectors

Sugarcane species-specific plastid transformation vector SOFM2 was developed for targeted insertions into the *trn*I (97663–98685)-*trn*A (98751–99633) inverted repeat region of the plastome (GenBank accession No. NC 006084). The cloned fragment served as flanking borders in the final transformation vector. These flanking sequences (*trn*I-*trn*A) were amplified from the sugarcane plastid genome by PCR, using primers P1 (5′-GAT ATC AAA ACC CGT CCT CAG TTC GGA TTG C-3′) and P2 (5′-GAT ATC CAC GAG TTG GAG ATA AGC GGA-3′). P1 primer annealed to nucleotides 97101–97126 and P2 annealed to 99620–99641 of the plastome. A DNA fragment carrying restriction sites for endonucleases: *Bcu*I, *Kpn*I, *Apa*I, *Xho*I, *Hinc*II, *Bsu*15I, *Hind*III, *Eco*321, *EcoR*I, *Pst*I, *Sma*I, *Bam*HI, *Bcu*I, *Xba*I, *Not*I, *Bstx*I, and *Sac*I, was amplified by primers P3 (5′-GGT ACC ACT AGT GGG CCC CCC CTC GA-3′) and P4 (5′-GAG CTC CAC CGC GGT GGC GGC CGC T-3′) and cloned in the intergenic region to facilitate further cloning. The selection marker FLARE-S (fluorescent antibiotic resistance enzyme, spectinomycin, and streptomycin) encoding a bifunctional protein obtained by translational fusion of the aminoglycoside 3’-adenyltransferase (*aadA*) gene with the green fluorescent protein (*gfp*) gene was cloned at *EcoR*I/*Hind*III sites.

### 4.4. Sugarcane Plastome Transformation

The optimized conditions for efficient calli induction and regeneration were used to engineer the *Saccharum* plastome. Among the evaluated genotypes, genotype HSF-240 was selected for transformation owing to its good response to callogenesis and regeneration. Calli were bombarded with SOFM2 plasmid DNA-coated gold particles of 0.6 μm diameter, using a Biolistic gun PDS-1000/He (Bio-Rad, Hercules CA, USA), following the transformation procedures described by Khan and Maliga [11]. The calli were incubated in the dark at 26 ± 1 °C for 48 h before transfer to selective medium supplemented with 350 mg/L of streptomycin sulfate (Phytotechnology, Lenexa, KS, USA). After two weeks of incubation on callus induction medium, antibiotic-resistant calli were shifted to selective RMSDBK medium. The streptomycin-resistant shoots were rooted on MS medium containing 4 mg/L IBA.

### 4.5. Tracking Fluorescent Protein (gfp) in Plastids/Cells

Streptomycin-resistant calli were initially inspected for fluorescence using a hand-held long-wave UV lamp. The proliferating embryos and leaf segments of the putative transgenic sugarcane plants were subjected to fluorescence microscopy using a stereomicroscope equipped with GFP detection (Olympus SZX America, Melville, NY, USA). Sub-cellular localization of GFP was verified by a laser-scanning confocal microscope (Sarastro 2000 Confocal Image System; Molecular Dynamics, Sunnyvale, CA, USA). GFP fluorescence was detected in the FITC (Fluorescein isothiocyanate) channel (488–514 nm) whereas chlorophyll fluorescence was detected in the TRITC (Tetramethyl rhodamine isothiocyanate) channel (560–580 nm).

### 4.6. Total Cellular DNA Extraction and PCR Analyses

Total cellular DNA was isolated from leaf tissues of antibiotic-resistant sugarcane clones as well as from nontransformed plants. PCR analysis was carried out to validate transgene integration. *Taq* DNA polymerase (Fermentas, Hanover, MD, USA) and platinum *Taq* polymerase (Fermentas, Hanover, MD, USA) were used with 100–300 ng of genomic DNA as a template. The *gfp* gene was amplified with primers P5 (5′-CCA TGG CTA GTA AAG GAG AA-3′) and P6 (5′-TTA TTT GTA TAG TTC ATC CA-3′). The left border fragment was amplified with primers P1 (5′-GAT ATC AAA ACC CGT CCT CAG TTC GGA TTG C-3′) and P8 (5′-GGG CTG ATA CTG GGC CGG CAG G-3′).

## 5. Conclusions

A proficient regeneration protocol was developed for elite sugarcane genotypes. Using the protocol, species-specific plastid transformation vector, cell-autonomous selection, and visual detection approaches, plastid transformation was achieved in sugarcane. Streptomycin-resistant clones remained heteroplasmic after selection and regeneration on streptomycin-containing medium, referring to the complex pattern of division and sorting of cells in the meristematic tissues as another important factor in the purification of transplastomes to homoplasmy in sugarcane.

## Data Availability

Not applicable.
